# The impact of protozoa addition on the survivability of *Bacillus* inoculants and soil microbiome dynamics

**DOI:** 10.1038/s43705-022-00166-9

**Published:** 2022-09-05

**Authors:** Panji Cahya Mawarda, Xavier Le Roux, Melissa Uribe Acosta, Jan Dirk van Elsas, Joana Falcão Salles

**Affiliations:** 1 Microbial Community Ecology Cluster, Expertise Group GREEN, Groningen Institute of Evolutionary Life Sciences (GELIFES), University of Groningen, Nijenborgh 7, 9747 AG Groningen, The Netherlands; 2 Research Center for Environment and Clean Technology, National Research and Innovation Agency Republic of Indonesia (BRIN), Komplek LIPI Bandung, Jalan Sangkuriang Gedung 50, Bandung, 40135 Indonesia; 3 Laboratoire d’Ecologie Microbienne, INRAE, CNRS, Université de Lyon, Université Lyon 1, UMR INRAE 1418, UMR CNRS 5557, 43 Boulevard du 11 Novembre 1918, 69622 Villeurbanne, France; 4 Plant-Microbe Interactions Group, Department of Biology, Faculty of Science, Utrecht University, Padualaan 8, 3584 CH Utrecht, The Netherlands; 5 Pollution Diagnostics and Control Group (GDCON), Biology Institute, University Research Campus (SIU), University of Antioquia (UdeA), Calle 70 No. 52-21, Medellín, Colombia

**Keywords:** Microbial communities, Soil microbiology, Microbial ecology, Molecular ecology

## Abstract

Protists’ selective predation of bacterial cells is an important regulator of soil microbiomes, which might influence the success of bacterial releases in soils. For instance, the survival and activity of introduced bacteria can be affected by selective grazing on resident communities or the inoculant, but this remains poorly understood. Here, we investigated the impact of the introduction in the soil of two protozoa species, *Rosculus terrestris* ECOP02 and/or *Cerocomonas lenta* ECOP01, on the survival of the inoculants *Bacillus mycoides* M2E15 (BM) or *B. pumilus* ECOB02 (BP). We also evaluated the impact of bacterial inoculation with or without protozoan addition on the abundance and diversity of native soil bacterial and protist communities. While the addition of both protozoa decreased the survival of BM, their presence contrarily increased the BP abundance. Protists’ selective predation governs the establishment of these bacterial inoculants via modifying the soil microbiome structure and the total bacterial abundance. In the BP experiment, the presence of the introduced protozoa altered the soil community structures and decreased soil bacterial abundance at the end of the experiment, favouring the invader survival. Meanwhile, the introduced protozoa did not modify the soil community structures in the BM experiment and reduced the BM + Protozoa inoculants’ effect on total soil bacterial abundance. Our study reinforces the view that, provided added protozoa do not feed preferentially on bacterial inoculants, their predatory behaviour can be used to steer the soil microbiome to improve the success of bacterial inoculations by reducing resource competition with the resident soil microbial communities.

## Introduction

Global food demand will soon outpace global crop production [1], as the current world population may reach 9.8 billion in 2050 [2]. Increasing food production has mainly been achieved through agricultural intensification, which has generated many environmental issues [3]. To counteract the environmental footprint of current agricultural practices, several countries are investing in the development of microbial inoculants [4] to boost crop productivity by reducing crop dependence on fertilisers and pesticides [5–7]. However, tailoring microbial inoculants to successful field application remains exceptionally challenging. One major limitation is the inability of many inoculants to sustain high population densities following introduction [8], as they must breach the often intense abiotic and biotic counterpressure from the soil microbiome [9, 10]. The mechanisms driving this counterpressure are often explained by resource competition and antagonism, which results in the low survivability of the inoculant [11–13]. However, other mechanisms controlling bacterial densities in soils, such as phage and protists activities, may affect the incoming and resident bacteria, potentially influencing the fate of introduced microbes. Although predation by protozoa (defined as heterotrophic protists) has been recognised as a “top-down” regulator steering the structure and function of soil microbiome [14, 15], this facet is rarely included in studies on the fate of microbial inoculants in soil.

Recently, the use of protozoa as inoculants, alone or in parallel with bacterial inoculants, has been proposed for several purposes. First, the bacterivorous features of protozoa can lead to nutrient releases, given that protozoan C:N ratios are often higher than those of their bacterial prey [16]. In the rhizosphere, protozoa can thus foster nutrient mineralisation, which benefits plants [17]. Moreover, protozoa can also act as biocontrol agents due to their predation on plant pathogens [18] and their secretion of extracellular compounds with bactericidal features [19]. They can also enhance plant immunity and hormonal balances [20, 21].

The combination of the plant beneficial effects of protozoan and bacterial inoculations might represent an interesting strategy to boost crop production. However, it is essential to evaluate to what extent the protozoa interfere with the survival of the introduced bacteria, either positively or negatively. Predation by protozoa is known to be often selective, i.e., it depends on the protozoan feeding mode and motility, the prey morphology (e.g., prey cell size and surface properties) and chemical traits (e.g., secondary metabolite secretion) [15]. Moreover, prey such as bacteria, fungi [18] and other protists [22] might possess defence mechanisms that can prevent detection, ingestion and digestion by protozoa [15]. Previous studies [23, 24] have shown that added protists could increase the survival of introduced bacteria in soil by activating their defensive secondary metabolites. However, other relevant mechanisms have remained underexplored. First, added protists can predate on the soil microbiome. This can (even in a transient manner) reduce the soil’s total bacterial abundance, improving the survival of the bacterial inoculant by alleviating competition, due to the removal of competitors and the release of nutrients. More generally, predation by added protists might decrease the abundance of predation-sensitive and increase predation-resistant taxa, thus modulating microbiome structure. This can then influence the survival of the bacterial inoculant, which is regulated by the soil microbiome composition [25, 26]. However, depending on the identity of the introduced bacterial strain and the protists, the latter might predate the bacterial inoculum, hence impairing its survival.

Here we evaluated whether the protozoan’s potential to “engineer” the abundance and composition of the soil microbiome could be used as a strategy to improve inoculant survival. We hypothesise that protozoan addition improves the survival of bacterial inoculants in soils, by releasing the resource competition between native and introduced bacterial species, despite the risk of direct predation on the bacterial inoculant. We selected two protozoan species, the plodding amoeba *Rosculus terrestris* ECOP02 (from now on referred to as *Rosculus*) and the free-swimming flagellate *Cercomonas* lenta ECOP01 (*Cercomonas*), for use in conjunction with the bacterial inoculants *Bacillus mycoides* M2E15 (from now on referred to as BM) or *B. pumilus* ECO-B-002 (BP). Each *Bacillus* strain was selected due to its ability to promote the plant growth, likely via phosphate solubilisation, iron acquisition, indole acetic acid and antimicrobial peptides (i.e., bacteriocin) production [27, 28]. Indeed, previous studies have shown that these *Bacillus* strains promoted the growth of sugar beet, cucurbits, tobacco and grass [27, 29–31]. Whereas the two protozoan species were chosen for different size and feeding strategies [32, 33], which determines their movement in the soil depending on the soil pore neck size [34, 35]. *Rosculus* moves using pseudopodia, feed via surface gliding phagocytosis, and have a smaller volume compared to *Cercomonas* [32, 36]. Meanwhile, *Cercomonas* move using flagellate, feed via flagellum-mediated filter feeding, and have a larger volume compared to *Rosculus* [32, 37]. Together with the aforementioned *Bacillus* features, we expect different characteristics between these two protists, would render different predatory impacts on soil microbiome dynamics and the *Bacillus* survival. The addition of these two protozoa into an organic fertiliser containing other *Bacillus* species favoured the latter’s persistence in soil [28].

We tested to what extent the survival of each introduced *Bacillus* strain was influenced by co-introduced *Rosculus* and/or *Cercomonas* in soil microcosms over 44 days. We also examined whether the impact of the protozoans might be explained by effects on the total soil bacterial abundance and the structure and composition of the soil bacterial and protist communities. Given previous results [28], we hypothesised that these protozoa would foster the survival of each of the two bacterial inoculants, particularly in relation to predation-induced modifications of the abundance and composition of the resident bacterial—and possibly protist—communities that compete with the *Bacillus* and protozoa inoculants.

## Materials and methods

### Soil and microcosm preparation

The soil was collected from a potato field (sandy loam, pH 4.75) in Leeuwarden, Friesland, The Netherlands. The soil was homogenised and sieved through a 2 mm mesh and adjusted to pH 7.0 by adding 2.33 g Ca(OH)_2_/kg soil to accommodate the survival of protozoa and bacterial inoculants, since they did not grow well under acidic condition. Soil microcosms were prepared by dividing 40-g portions of this soil and placing them inside sterile glass jars capped by sterile aluminium foil. Soil moisture was kept constant at 65% of the water holding capacity (WHC) by regularly replenishing the water until bacterial and protozoa invaders were introduced. The soil was incubated for 2 weeks at room temperature to allow the re-establishment of the soil microbiome in all microcosms.

### Experimental design and inoculation approach

**Fig. 1:**
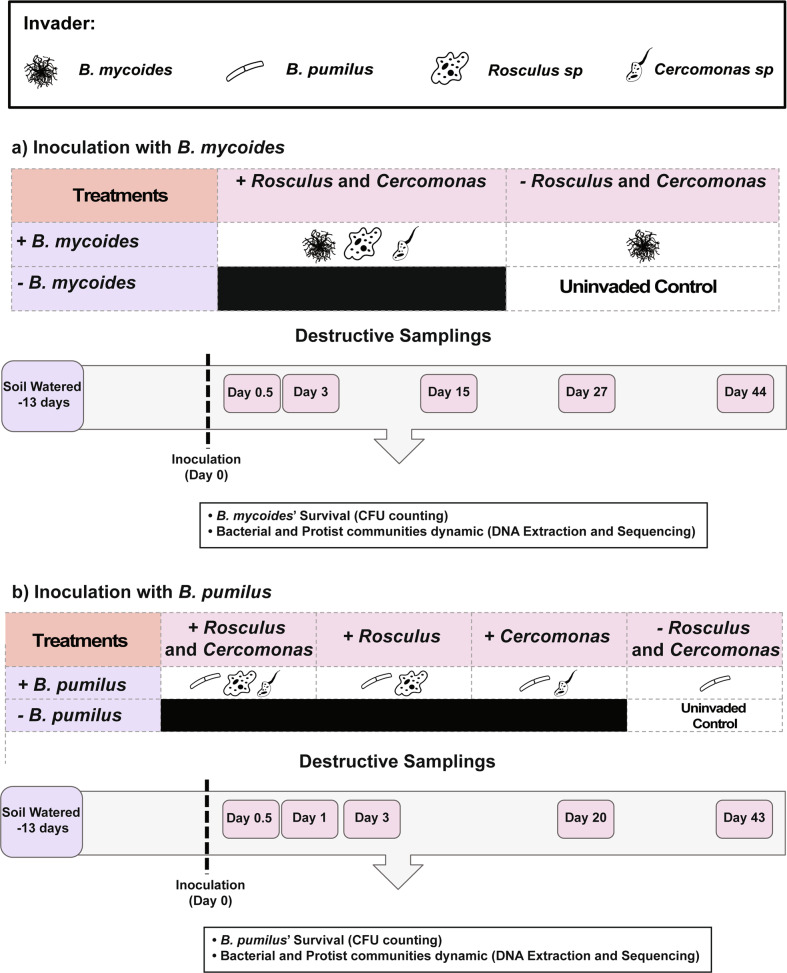
Summary of the experimental design for the experiments. (**a**) inoculated by *Bacillus mycoides* M2E15 and (**b**) *B. pumilus* ECO-B-02, with or without protozoa.

The treatments were created by inoculating the microcosms with different combinations of bacterial (BM and BP) and protozoan invaders (*Rosculus* and *Cercomonas*). At the same time, control was prepared by adding sterile water (Fig. 1). For the BM experiment, the treatments consist of microcosms inoculated by BM alone; BM*, Rosculus*, and Cercomonas altogether (BM + R + C); and non-inoculated control (Fig. 1a). In the BP experiment, the treatments consist of microcosms inoculated by BP alone; BP, *Rosculus*, and *Cercomonas* (BP + R + C); BP and *Rosculus* (BP + R); BP and *Cercomonas* (BP + C); and non-inoculated control (Fig. 1b). Destructive samplings were made on days 0, 3, 15, 27, and 44 for the BM experiment and days 0, 1, 3, 20, and 43 for the BP experiment, using triplicates for each treatment and sampling date. This, hence, comprised 45 microcosms for BM (3 treatments × 5 sampling dates × 3 replicates) and 75 microcosms for BP (5 treatments × 5 sampling dates × 3 replicates). We conducted two separated experiments with different sampling timepoints based on the inoculant survival patterns generated from our previous study [26].

Rifampicin-resistant strains of BM and BP were developed by a spontaneous mutation, which did not influence the fitness of the mutated strains compared to that of the wild-type strains [26]. Both strains were grown in LB broth overnight at 28 °C. The cultures were then washed three times with 0.85% NaCl solution via centrifugation at 6000 rpm for 3 min. The cell pellets from each culture were resuspended to reach an OD = 0.8 at 590 nm in 1 ml of saline solution, which gave a concentration of 7.5 × 10^6^ CFU/g of soil for BP and 7.4 × 10^6^ CFU/g of soil for BM. Meanwhile, the protozoa cell suspensions of *Rosculus* and *Cercomonas* were prepared in the concentration of 10^5^ cell/g of soil via cell counting. These protozoa were cultivated using *E. coli* as their food source. In each experiment, the ratio between each or both protozoa and *Bacillus* was adjusted to 1:1000 via dilution and cell counting, after which the resulting cell suspensions were used for soil microcosms inoculation. Inoculations increased soil moisture in each microcosm from 65 to 75% of WHC, which was then kept constant by replenishing the water until the end of the experiment.

### Monitoring of the survival of *Bacillus*—total CFU and spores

The survival of *Bacillus* was tracked by 1:10 serial dilution plating on TSA medium containing rifampicin (50 µg/ml) and cycloheximide (400 µg/ml). The agar plates were incubated at 28 °C for 24 h for BM and 48 h for BP. *Bacillus’* spore counts were enumerated by heating the diluted samples at 80 °C for 20 min and plated on TSA containing rifampicin and cycloheximide as described above.

In addition, the soil bacterial abundance was also quantified via quantitative PCR targeting the V4 variable region of the *16S rRNA* gene (see Supplementary Document 1 for detailed protocol).

### Soil DNA extraction, illumina sequencing, and processing

Total DNA was extracted from 0.5 g of soil at each sampling date using the DNeasy Powersoil Kit (Qiagen, Hilden, Germany) according to the manufacturer’s instructions. Genomic DNA concentration was quantified using NanoDrop 2000 spectrophotometer (Thermo Scientific, USA) and adjusted to 30–90 ng/µl. Each extracted DNA sample was then sent for bacterial *16S rRNA* gene sequencing targeting the V4 hypervariable regions (forward primer 16S-515F: 5’-GTGCCAGCMGCCGCGGTAA-3’; reverse primer 16S-806R: 5’-GGACTACHVGGGTWTCTAAT-3’) and for protist *18S rRNA gene* sequencing targeting the V9 hypervariable regions (forward primer 18S-1391F: 5’-GTACACACCGCCCGTC-3’; reverse primer 18S-EukBr: 5’-TGATCCTTCTGCAGGTTCACCTAC-3’). The sequencing was done on Illumina Miseq 2 × 300 base paired-end reads (Illumina, San Diego, California) at the University of Minnesota Genomic Centre (UMGC) (Minneapolis, MN, USA), using their dual indexing method [38].

The analysis of these raw sequences was then processed in Quantitative Insights Into Microbial Ecology (QIIME 2, https://qiime2.org). The adaptors and primer sequences were already removed and assembled for each sample according to their unique barcode by the UMGC. The split sequences for each sample were paired-end merged, denoised, dereplicated, and trimmed using DADA2 [39]. This tool also filters chimeric sequences and removes primer sequences and singleton. For bacterial *16S rRNA* gene sequences, DADA2 trimmed the sequence at positions 254 bp for forward and 209/210 bp for the reverse reads, for the BM and BP experiment, respectively. For protist *18S rRNA* gene sequences, DADA2 trimmed them at positions 212 bp forward and 187 bp reverse in the BM experiment, whereas in the BP experiment, the sequences were trimmed at 212 bp and 186 bp for forward and reverse reads, respectively. For both bacterial and protist sequences, the expected error for forward and reverse reads was set at five, and the minimum overlap in paired-end merging was set at 20 bases.

After being processed in DADA2, the sequences were assigned to Amplicon Sequence Variants (ASVs)—provided in the feature table and the representative sequences—with a 99% identity level threshold. Representative sequence sets were aligned by MAFFT (ver. 7) [40] from which a phylogenetic tree was created using FastTree (ver. 2.1) [41]. The representative sequences of bacterial *16S rRNA* gene were classified using the Greengenes taxonomy via the Ribosomal Database Project classifier [42]. In contrast, the representative sequences of the protist *18S rRNA* gene were matched against Silva (ver 132) [43], both at a nucleotide sequence similarity of 99%. The Naïve Bayesian classifier was trained on the Greengenes 13_8 99%, and the Silva 18S_99_SILVA132_RL250 database using forward and reverse primers used to amplify the V4 region of *16S rRNA* gene and V9 region of *18S rRNA* gene, respectively.

### Statistical analyses

The soil bacterial and protist community structures were analysed in R 1.2.5019 using the Vegan and Phyloseq packages. The ASVs were subjected to rarefaction using phyloseq::rarefy function to minimise the sampling effects. The feature tables were rarefied for bacterial 16S rRNA gene sequences at 6,236 sequences and 6,106 sequences per sample for the BM and BP experiment, respectively. For protist *18S rRNA* gene sequences, the feature table in the BM experiment was rarefied at 9690 sequences, whereas in BP, the rarefaction was done at 6237 sequences.

We removed *16S rRNA* gene sequences belonging to mitochondria, chloroplast, archaea, and unidentified kingdom. For *18S rRNA* gene sequences, those belonging to fungi, plants, animals, unknown kingdom, and unidentified phylum were removed. To eliminate false-positive eukaryotic species further, sequences affiliated with phylum *Opisthokonta*, Order *metazoa*, order *Charophyta*, and class *Rhodophyceae* were also excluded.

The BM experiment generated a dataset containing 8986 ASVs distributed over 43 samples for *16S rRNA* gene sequences and 14,131 ASVS distributed over 43 samples for *18S rRNA gene* sequences. For the microcosms invaded with BP, the dataset contained 15,101 ASVs distributed over 70 samples for *16S rRNA* gene sequences and 28,192 ASVS distributed over 74 samples for *18S rRNA gene* sequences. These data were used to quantify the variation in soil protist and bacterial communities’ structure based on unweighted Unifrac distance [44].

To evaluate treatment effects at each date on the abundances of each bacterial inoculant, inoculant spores, and total soil bacteria, Kruskal–Wallis and Wilcox’s post hoc were carried out for non-parametric data. Whereas ANOVA and post hoc Tukey Nemeyi tests were carried out for parametric data. We visualised treatment effects on community structures via principle coordinates analysis, for which permutational multivariate analysis of variance (PERMANOVA) was used to evaluate significant differences between treatments.

## Results

**Fig. 2:**
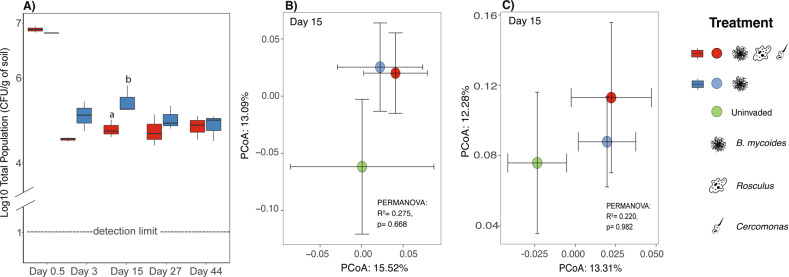
Survival of *Bacillus mycoides* M2E15 and the impact of inoculation treatments on the soil bacterial and protist communities structure. (**A**) Survival of *B. mycoides* M2E15 in the presence and absence of *Rosculus terrestris* ECOP02 and *Cercomonas lenta* ECOP01 over time. Values represent the log CFU of the population per gram of soil. Bars represent the standard error of the mean. Inoculation treatment affects the soil bacterial (**B**) and protist (**C**) communities. Centroids for each treatment are shown along with their standard errors (error bars).

To examine the impact of protozoan presence on the survival of the *Bacillus* inoculants, we monitored the latter’s abundance (total and spore populations, log CFU/g of soil) in soil microcosms with or without added protozoa for up to 44 days following their release (Fig. 2A). We observed different survival patterns for each *Bacillus* strain in the presence versus the absence of the protozoa, and other effects of the inoculants on the soil microbiome, as detailed in the following.

### BM experiment—survival of the introduced populations

The addition of *Rosculus* and *Cercomonas* to systems containing the BM inoculant led to lower survival of the bacterial invader (both for total and spore populations) compared to inoculation with BM alone at day 15 post-inoculation (p.i) (total population: Fig. 2A, *t*-test, *p* < 0.05; total spores: Fig. S1, *t*-test, *p* < 0.05).

Moreover, time significantly affected the BM population dynamics (ANOVA, *p*_(time)_ = 0.008). The bacterial population densities initially declined rapidly, from an initial 6.8 log CFU/g soil to 4–5 log CFU/g soil at day 3 p.i, at which level these stabilised (Fig. 2A). Conversely, the BM spore population sizes significantly increased, from 0.5 log CFU/g soil to around 4–5 log CFU/g soil in all treatments at day 3 p.i. (Fig. S1a, ANOVA, *p*_(time)_ = 0.007).

### BM experiment—impact on the native soil community structure and total bacterial abundance

**Fig. 3:**
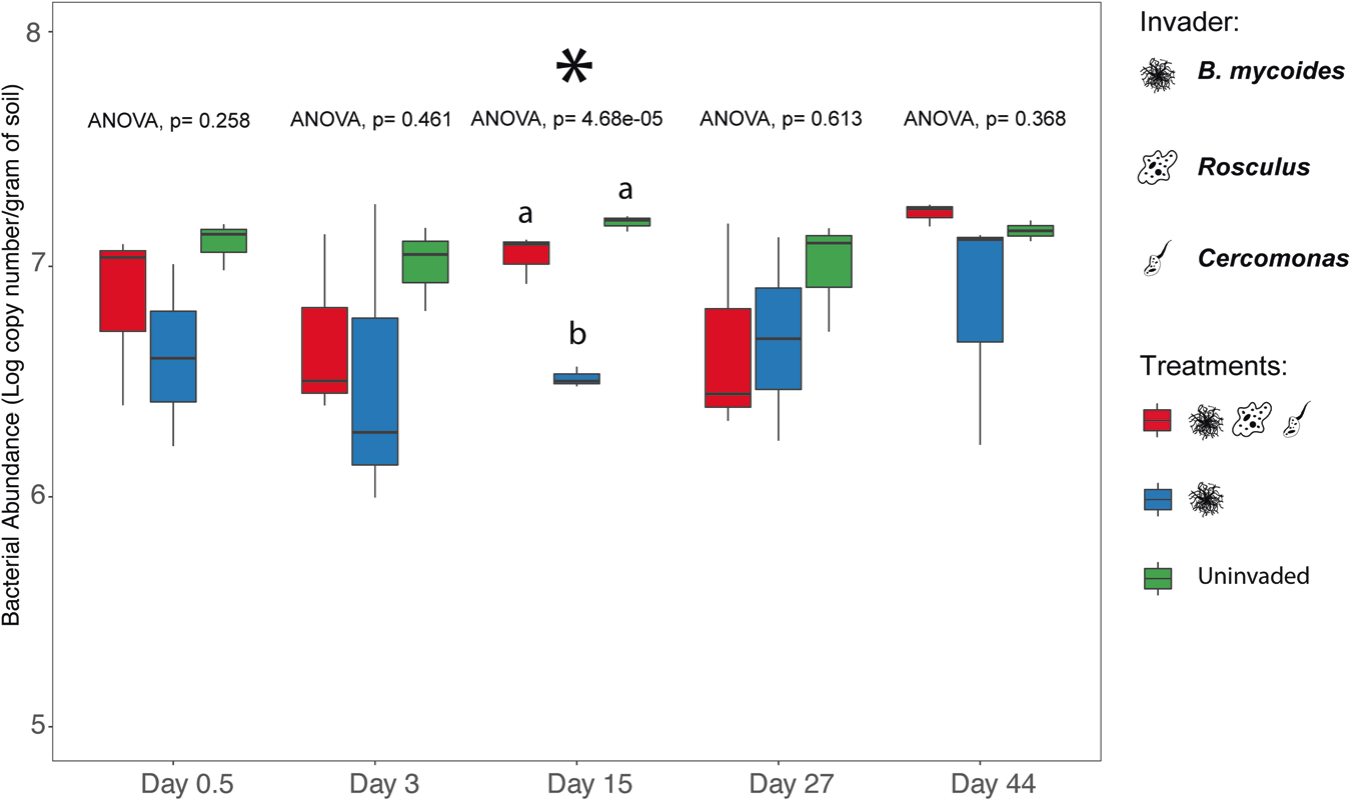
Temporal dynamics of the total soil bacterial abundance between treatments in the *Bacillus mycoides* experiment. Values represent qPCR results in the log of 16S rRNA gene copies per gram of soil. Bars represent the standard error of the mean.

To examine the inoculant effect on the soil bacterial and protist community structures, we calculated the unweighted Unifrac distances (phylogeny-based) on each sampling day across treatments and control (Fig. S2). High resistance to invasion impacts was observed from the bacterial and protistan community structures measured throughout the experiment (Fig. S2a, b). Specifically, at day 15 p.i, when the survival of BM was higher in the absence of protozoa, the presence of *Rosculus* and *Cercomas* did not shift the structures of the bacterial (Fig. 2B, PERMANOVA, *p* = 0.67) and protist communities (Fig. 2C, PERMANOVA, *p* = 0.50) away from those of the (uninvaded) control. However, at day 15 p.i, the invasion by BM caused significant changes in bacterial community abundance (Fig. 3, ANOVA, *p* < 0.0001). The invasion by BM alone significantly reduced the total bacterial abundance compared to the uninvaded control from 7.2 log copy number/g soil to 6.5 log copy number/g soil (Tukey’s post hoc, *p* < 0.001). At the same time, the presence of protozoa did not change this abundance compared to the control (Tukey’s post hoc, *p* > 0.05).

### BP experiment—survival of the bacterial Inoculant

**Fig. 4:**
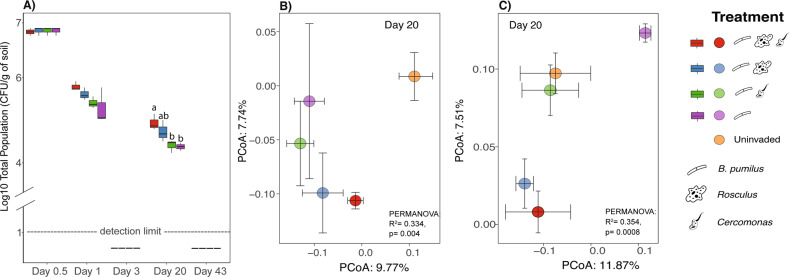
Survival of *Bacillus pumilus* ECOB02 and the impact of inoculation treatments on the soil bacterial and protist communities structure. (**A**) Survival of *Bacillus pumilus* ECOB02  in the presence and absence of *Rosculus terrestris* ECOP02 and *Cercomonas lenta* ECOP01 over time. Values represent the log CFU of the population per gram of soil. Bars represent the standard error of the mean. Inoculation treatment affects the soil bacterial (**B**) and protist (**C**) communities. Centroids for each treatment are shown along with their standard errors (error bars).

Remarkably, the addition of both *Rosculus* and *Cercomonas* in the BP experiment enhanced the levels of culturable BP compared to the invasion by BP + C and BP alone at Day 20 p.i (Fig. 4A, ANOVA, *p* < 0.01, Tukey’s post hoc, *p* < 0.05). Although the total population size of BP was higher when *Rosculus* was added than BP + C and BP alone, this was not significant, as it turned out to be statistically similar across the treatments (Tukey’s post hoc, *p* > 0.05). Moreover, the BP spore populations remained below the detection limit throughout the experiment in all treatments.

Time significantly affected the population sizes of BP, with the platable inoculant populations dropping below the detection limit (1 log CFU/g soil) at day 3 p.i (Fig. 4A). However, platable BP populations reappeared, at around 4–5 log CFU/g soil, at day 20 p.i, after which they dropped to below the detection limit at day 43 p.i. in all treatments (Fig. 4A). We cannot easily explain this finding. However, it is known that introduced cells may pass through periods in which they do not readily form colonies on isolation plates, a phenomenon called the viable-but-nonculturable conundrum [45].

### BP experiment—impact on the native soil community structure and total bacterial abundance

The release of BP + R + C, BP + R, and BP + C in the BP experiment significantly impacted the soil bacterial and protist communities (Fig. S3a, b). Regarding the bacterial community, the invasion changed the community structures on day 20 p.i (Fig. 4B, PERMANOVA, *R*^2^ = 0.33, *p* = 0.004) and day 43 p.i (Fig. S3a, day 43, *R*^2^ = 0.36, *p* = 0.028) compared to the uninvaded control. At day 20 p.i, when the presence of *Rosculus* and *Cercomonas* increased the survival of BP, the BP + R + C and BP + R treatments shifted the structures of the bacterial communities away from those of the uninvaded systems (Fig. 4B, pairwise-Adonis, *p* < 0.05). Conversely, the bacterial communities in the BP + C and BP treatments clustered together and were not significantly different from those in the uninvaded ones (pairwise-Adonis, *p* > 0.05). At day 43 p.i, the BP + C treatment shifted the bacterial community structure away from the uninvaded control (Fig. S3a, day 43, pairwise-Adonis, *p* < 0.05). The taxonomic information on the most affected bacterial communities due to these inoculations can be found in Supplementary Table 1.

Regarding the protist community structures, we observed significant effects of invasion on different days, i.e. day 3 (Fig. S3b, PERMANOVA, *R*^2^ = 0.32, *p* = 0.008), day 20 (Fig. 4C, PERMANOVA, *R*^2^ = 0.35, *p* = 0.0008), and day 43 p.i (Fig. S3b, PERMANOVA, *R*^2^ = 0.34, *p* = 0.002). Specifically, at day 20 p.i, the invasion by BP + R + C and BP + R altered the protist community structures away from those in the uninvaded control (pairwise-Adonis, *p* < 0.05). Meanwhile, the protist communities invaded by BP + C, BP alone and uninvaded controls clustered together (pairwise-Adonis, *p* > 0.05). At day 3 p.i (Fig. S3b), the invasion by BP alone altered the protist community structure away from the control (pairwise-Adonis, *p* < 0.05). In addition, on day 43 p.i (Fig. S3b), the addition of protozoa to all BP inoculant treatments (BP + R + C, BP + R, BP + C) changed the protist community structures away from the community invaded by BP alone and the control (pairwise-Adonis, *p* < 0.05). The taxonomic information on the most affected protists communities due to these inoculations can be found in Supplementary Table 2.

**Fig. 5:**
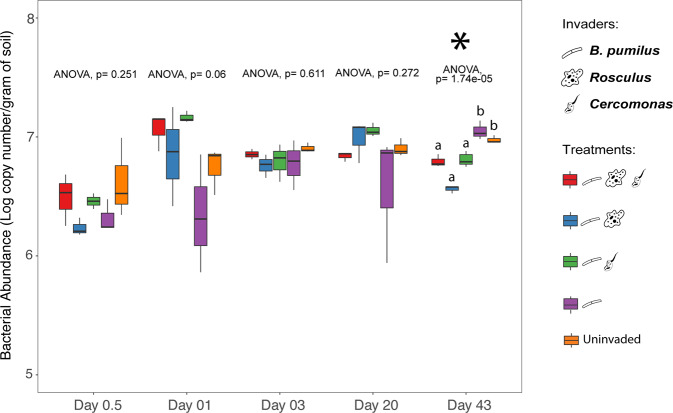
Temporal dynamics of the total soil bacterial abundance between treatments in the *Bacillus pumilus* experiment. Values represent qPCR results in the log of 16S rRNA gene copies per gram of soil. Bars represent the standard error of the mean.

The microbial invasion in the BP experiment also led to significant changes in the total bacterial abundance at day 43 p.i (Fig. 5: ANOVA, *p* < 0.0001). At this date, the presence of protozoa (BP + R + C, BP + R, and BP + C) significantly reduced the total bacterial abundance compared to the BP alone and uninvaded control treatments (Tukey’s post hoc, *p* < 0.001). The total bacterial abundance was similar for the BP alone and control treatments (Tukey’s post hoc, *p* > 0.05).

## Discussion

The potential of protists as soil inoculants lies in their ability to exert control on the bacterial prey they encounter in the soil. Increased availability of nutrients resulting from the microbial loop induced by these protists and the changes they generate in the resident microbiome community structure and composition may lead to enhanced survival of introduced bacteria. Here, we selected two protozoan species with reported beneficial activities for plant performance, i.e. *Rosculus* and *Cercomonas*, to address their effects on the establishment and survival of the bacterial inoculants BM and BP. Furthermore, the impact on the soil bacterial and protistan communities of the releases of BM and BP in the presence or absence of protozoa was evaluated.

### Presumed selective predation by the protozoa on the bacterial inoculant rather than the soil resident community likely decreases BM survival

Remarkably, we found that the added *Rosculus* and *Cercomonas* differentially impacted the population dynamics of the bacterial invaders, i.e., the impact depended on the *Bacillus* strain. In the BM experiment, the absence of protozoa resulted in increased invader survival. In contrast, *Rosculus* and *Cercomonas* favoured the establishment of BP. Consistently, in previous work, the impact of protozoan predators (*Cercomonas* spp, *Naegleria* spp, *Acanthamoeba* sp, *Vanella* sp) varied depending on the bacterial prey (*Pseudomonas* spp), even within the same bacterial genus [46]. Glücksman et al. [47] further showed that even closely-related and morphologically similar protists could have a different impact on soil bacterial communities. Indeed, preferential feeding habits of protists and their species-specific interactions have been reported in several studies [48, 49]. Our results strongly suggest that *Rosculus* and *Cercomonas* can, to some extent, prey on the released BM cells, decreasing survival. However, indirect effects mediated by resource competition (i.e an increase of predation-resistant bacterial communities after protozoa addition, which compete with the bacterial inoculants) could also reduce the BM survival.

In contrast, BM’s invasion without added protozoa negatively affected the total soil bacterial abundance, which may facilitate its survival. This is the first report where the inoculation of *Bacillus* decreased the total soil bacterial abundance, suggesting that BM was able to compete with the resident soil community. This finding is remarkable since introduced inoculants generally cannot stand the competition from resident ones that are established and have found their niche, even with the same/higher inoculant density than in our study [11, 25, 50]. However, it has been reported that resident bacteria that utilise a similar niche as a bacterial invader may suffer from competition for resources with the invader. Hence, their abundance may drop, allowing the microbial invader to conquer niche space at the expense of the resident microbiome niche occupant [51, 52]. Moreover, the BM used in our study is capable of producing Bacteriocin [27], a group of antibiotic compounds which inhibits a range of soil-bacteria [53]. This also could be the reason why BM significantly reduced the total bacterial abundance when inoculated alone.

### Selective predation by protozoa on resident soil bacteria improves BP Survival

In the BP experiment, the presence of *Rosculus* and *Cercomonas* favoured the establishment of BP. Indeed, previous in vitro study by Oosterkamp and Loznik [28] showed that the protozoa strains used in this study preferred to prey on *E. coli* than *B. pumilus* and *B. amyloliquefaciens*. The potential mechanisms driving this positive effect could be due to the modifications induced on the resident microbial communities, but a direct stimulatory effect via activation of bacterial defensive secondary metabolites might also be possible [23]. Our results suggest that the protozoa preferentially preyed on the native soil bacteria rather than the introduced bacteria, which altered the bacterial community structure by decreasing the abundances of the bacterial taxa sensitive to predation. If the sensitive taxa comprised individuals that are metabolic similar to the invader (i.e., exploit the same resources), their reduction would decrease competition and may have reinforced the reshaping of the soil community via competition, as shown by [25, 51]. As a consequence, the changes in the soil bacterial community structure may have indirectly contributed to shifting the protist community’s structure and their bacterial prey structure and composition. Such a link between bacterial and protist community structures has previously been reported by [29] and [54]. Another possible indirect effect could be associated with the decrease of resident protist communities that prey on the BP after protozoa release, imposed by competition and or antagonism by the invaders. In addition, these changes in the soil protist community may also result from direct competition for resources between the introduced protozoa and the resident protists.

A previous study showed that added *Acanthamoeba castellanii* improved the survival of the inoculant *Pseudomonas fluorescens* [23]. However, this was attributed to the production of secondary metabolites protecting the inoculant from predation. Here, the results suggest that selective feeding by protozoa altered the total bacterial abundance and resident community structure and modulated the “balance” across the populations making up the soil bacteriomes. Amacker et al. [39] indicated that future studies, including a detailed analysis of protist traits, their morphotype and taxonomy, could foster our understanding of such trophic interactions.

### Inoculant traits link to the impact on soil native communities

Apparent differences were found between the BM and BP experiments. In the BM experiment, the microbial release did not change the structure of the bacterial and protist communities. However, in the BP experiment, each invader incited significant changes in the tested communities, although this depended on the time point and the type of invaded community (protist/bacterial) being examined. Thus, the impact is linked to the type of invader and the interaction between protozoan and bacterial inoculants. Indeed, Gao et al. [32] showed that the amoeba *Rosculus* and the flagellate *Cercomonas* used in this study have different morphotypes and volumes, driving the physical scope of their effects. The study found that protist cell volume could explain a significant portion of the predation effects. Another study revealed that the cell volumes of cercozoan species link to predation activity [47]. According to macroecological theory, predator body size can serve as a proxy for feeding capacity and influence predation preference [55, 56]. Moreover, the size of protists determines their movement in the soil, depending on the soil pore neck size, which influences their grazing activities [34, 35]. It is also important to mention that in the field, most of the resident microbiome are shielded as microcolonies in undisturbed soils [57]. However, this shielding is potentially quite limited in disturbed systems, as in this lab experiment. Therefore, the added protozoa have likely acted more easily on resident organisms.

We also found that the addition of protozoa and bacteria led to a more substantial impact than the single-strain introduction of BP since the latter only temporarily affected the protist community structure. This may indicate that co-inoculation increases the magnitude of disturbance and selection pressure. A previous study suggested that the magnitude and frequency of a disturbance are key facets determining impact [58]. Moreover, Wang et al. [59] found that co-inoculation had a different effect on native soil communities than single-strain inoculation, especially for plant growth and soil nutrient mobilisation.

## Conclusion

Taken altogether, our results show that top-down control by protists, through selective predation, can be used to steer the soil microbiome to improve the success of bacterial releases into the soil. However, we demonstrate that a positive effect of the added protozoa on the bacterial inoculant survival can sometimes be jeopardised by preferential feeding of these protozoa on the incoming bacteria. As the impact was dependent on the protozoan species, a systematic characterisation of their functional traits, their ecological behaviour (e.g., their possibilities for movement in the soil) and feeding preferences of the protozoa are needed to predict better the consequences of their predation on the soil microbiome and introduced bacteria.

## Supplementary information

Supplementary Information

## Data Availability

Raw sequences were submitted to the NCBI Sequence Read Archive (SRA) and are available under the BioProject ID PRJNA830501.
