# Supplementary material for: The impact of protozoa addition on the survivability of Bacillus inoculants and soil microbiome dynamics
Source: ISME Commun. 2022 Sep 5;2:82. doi: 10.1038/s43705-022-00166-9 (PMC9723691; doi:10.1038/s43705-022-00166-9)
Supplement: Supplementary file 1 — Supplementary Information [file 43705_2022_166_MOESM1_ESM.pdf]

Supplementary Information

The Impact of Protozoa Addition on the Survivability of *Bacillus* inoculants and Soil Microbiome Dynamics

Panji Cahya Mawarda[1,2], Xavier Le Roux[3], Melissa Uribe Accosta[4,5], Jan Dirk van Elsas[1], Joana Falcao Salles[1]

[1] Microbial Community Ecology Cluster, expertise group GREEN, Groningen Institute of Evolutionary Life Sciences (GELIFES), University of Groningen, Nijenborgh 7, 9747 AG, Groningen, the Netherlands, Nijenborgh 7, 9747 AG, Groningen, The Netherlands.

[2]Research Center for Environment and Clean Technology, National Research and Innovation Agency Republic of Indonesia (BRIN), Komplek LIPI Bandung, Jalan Sangkuriang Gedung 50, Bandung 40135, Indonesia.

[3]Laboratoire d'Ecologie Microbienne, INRAE, CNRS, Université de Lyon, Université Lyon 1, UMR INRAE 1418, UMR CNRS 5557, 43 boulevard du 11 novembre 1918, Villeurbanne 69622, France.

[4]Plant-Microbe Interactions Group, Department of Biology, Faculty of Science, Utrecht University, Padualaan 8, 3584 CH, Utrecht, The Netherlands.

[5]Pollution Diagnostics and Control Group (GDCON), Biology Institute, University Research Campus (SIU), University of Antioquia (UdeA), Calle 70 No. 52-21, Medellín, Colombia.

*Corresponding author – Email: p.c.mawarda@rug.nl / panji.cahya.mawarda@brin.go.id

Supplementary Document 1- Quantitative PCR Protocol

Total DNA was extracted from 0.5 g of soil at each sampling date, using the DNeasy Powersoil Kit (Qiagen, Hilden, Germany) according to the manufacturer's instructions. DNA concentration was quantified using PicoGreen dsDNA assay (Invitrogen, Paisley, UK). The V4 region of the 16S rRNA gene was amplified using forward primer 16S-515F: 5'-GTGCCAGCMGCCGCGGTAA-3' and reverse primer 16S-806R: 5'-GGACTACHVGGGTWTCTAAT-3'. For each reaction, 12.5 µl of Power SYBRgreen master mix (Applied Biosystems, Carlsbad, California, USA) was added with 0.75 µl of each primer, 10 µl of ultrapure water, and 1 µl of template DNA. The cycling program began with a 10-minute denaturation phase at 95°C, followed by 40 cycles of denaturation at 95°C for 27 seconds, annealing at 62°C for 1 min, and extension at 72°C for 30 seconds. Quantification was carried out in the ABI Prism 7300 Cycler (Applied Biosystems, Foster City, California, USA). The standard curve came from plasmid DNA, where the 16S rRNA gene from Serratia plymuthica was cloned into the pGEM-T plasmid was used as a standard template with concentrations ranging from 10^6 to 10^1 copies / µl.

Figure S1. Spore Survival of *Bacillus mycoides* M2E15, BM, (A), and *B. pumilus* ECO-B-02, BP (B) in the presence and absence of *Rosculus* and/or *Cercomonas* over time. Values represent the log cfu of total spores per gram of soil. Bars represent the standard error of the mean.

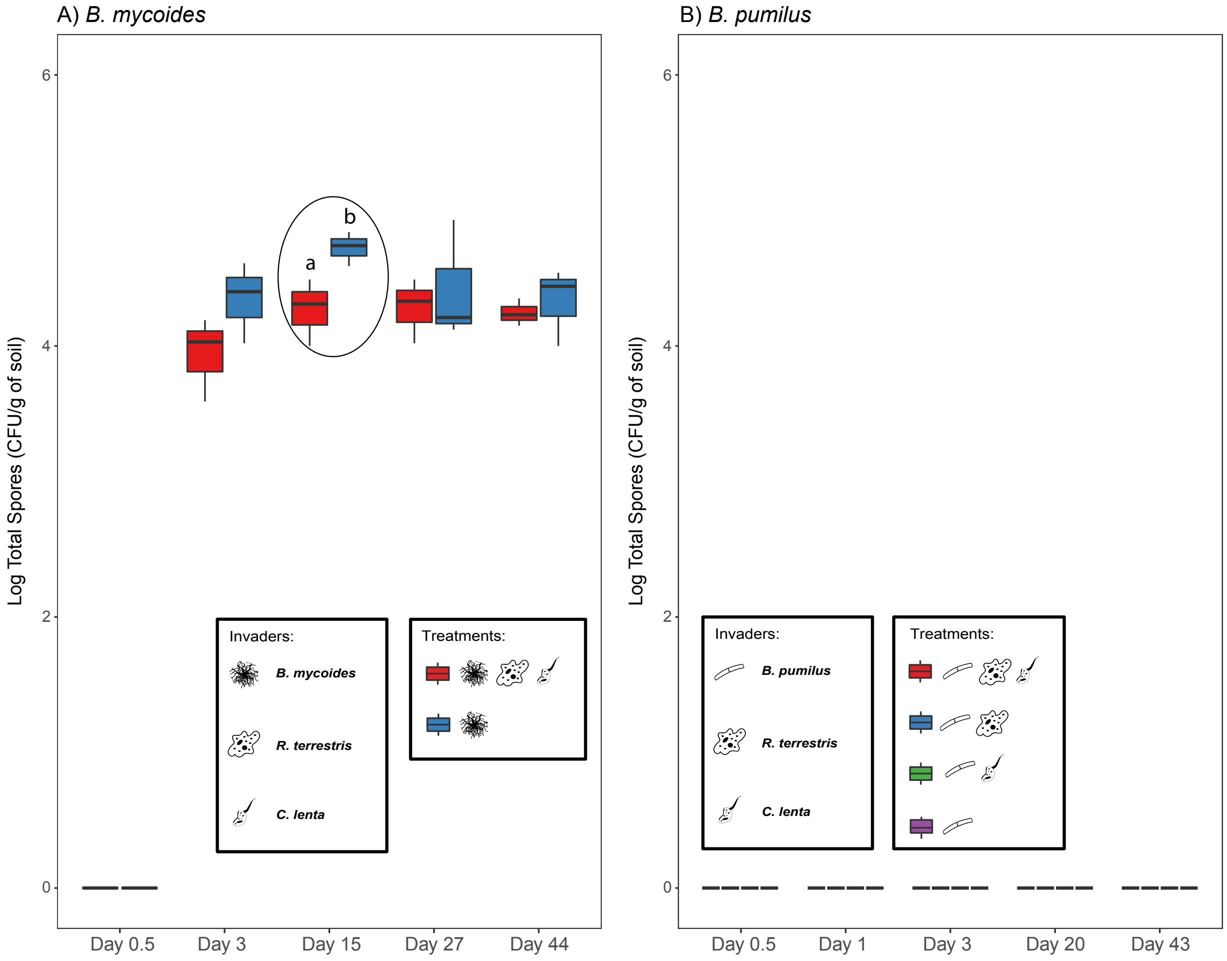

Figure S2. Variation of community structure in the *Bacillus mycoides* experiment. PCoA plot of unweighted unifrac distances over time, between each treatment for (a) bacterial and (b) protist communities. Centroids for each sampling time are shown along with their standard errors (error bars).

a) Bacterial community

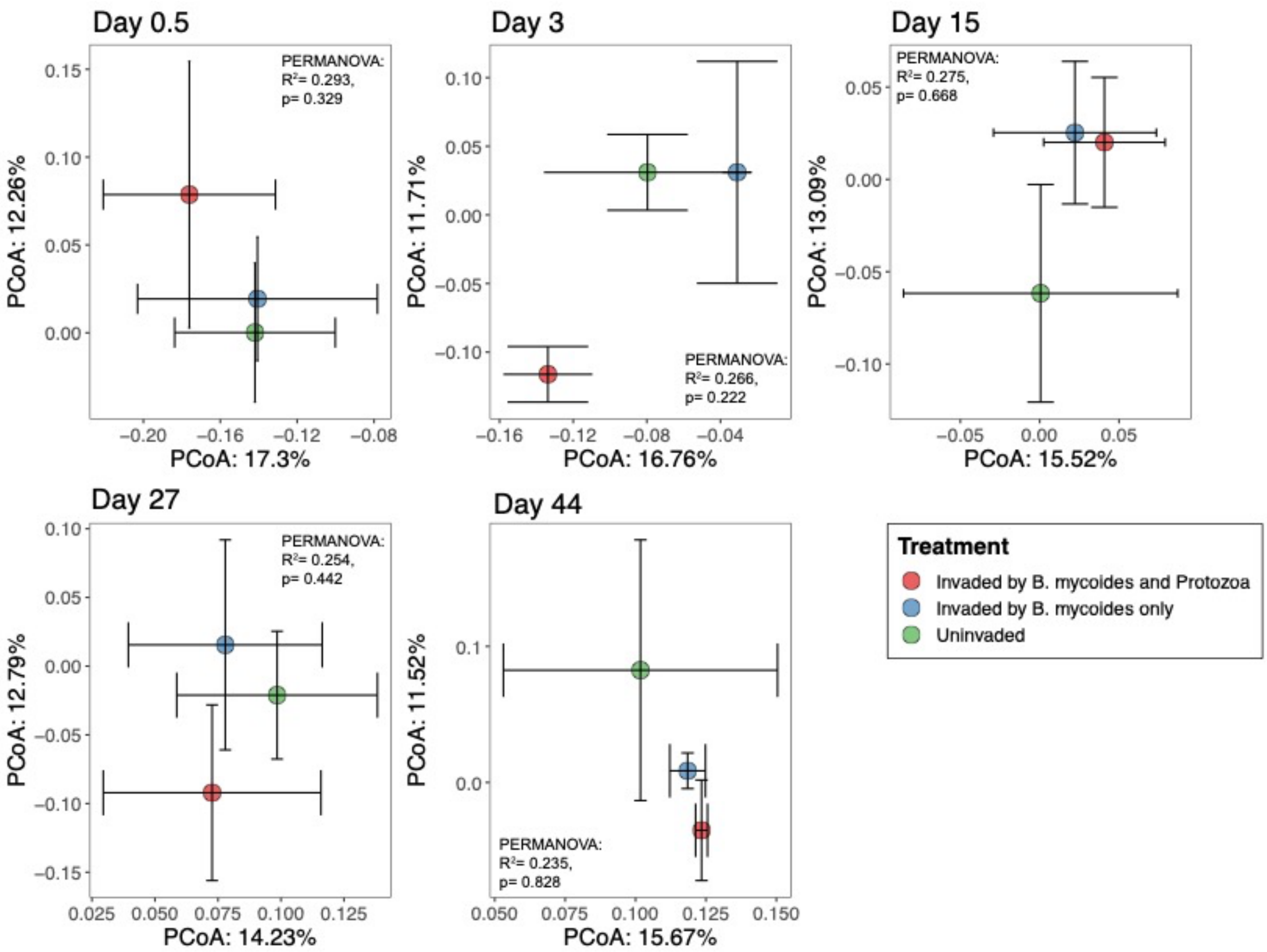

b) Protist community

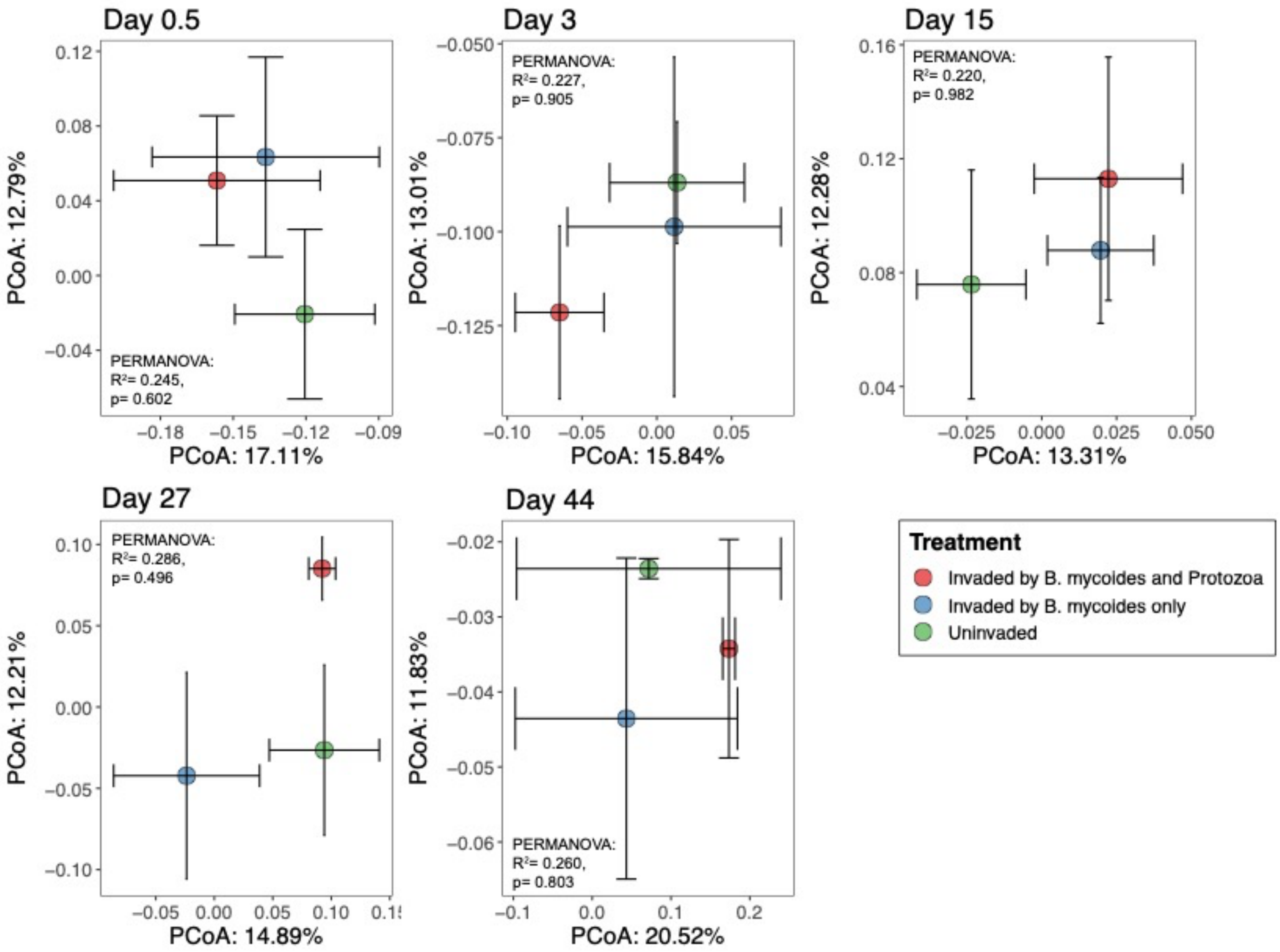

Figure S3. Variation of community structure in the *Bacillus pumilus* experiment. PCoA plot of unweighted unifrac distances over time, between each treatment for (a) bacterial and (b) protist communities. Centroids for each sampling time are shown along with their standard errors (error bars). Dashed circle with different colors indicates significant differences between treatments.

a) Bacterial community

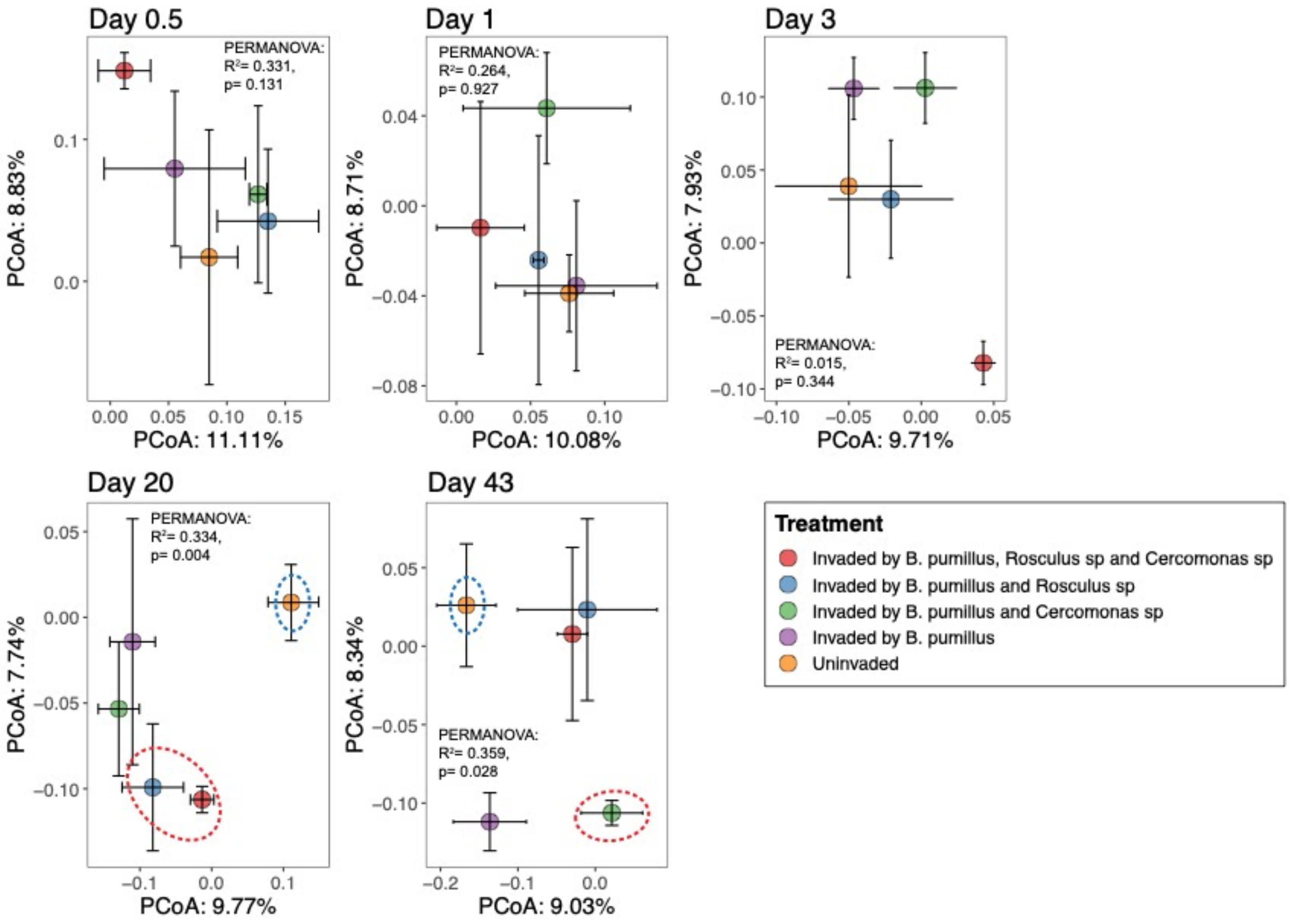

b) Protist community

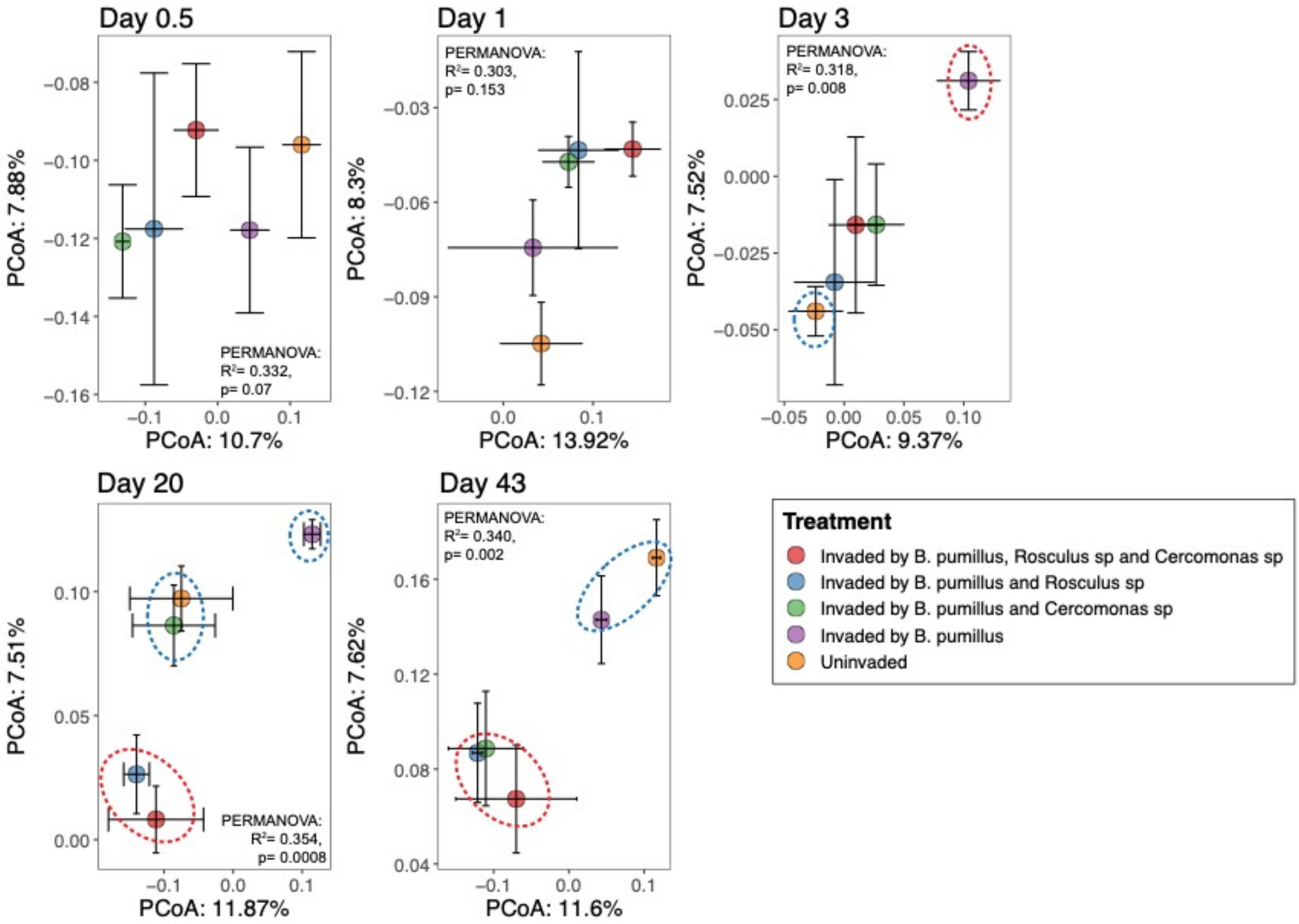

Supplementary Table 1

a) The most affected bacterial communities between BP+R+C and control at Day 20 post inoculation in the BP experiment

Kingdom	Phylum	Class	Order	Family	Genus	Species	Confidence	Av. dissim	Contrib. %	Cumulative %	Mean BP+R+C	Mean control
Bacteria	Firmicutes	Bacilli	Bacillales	Planococcaceae	Sporosarcina	Unidentified	0.758025	0.5386	0.5729	0.5729	18.3	64.3
Bacteria	Planctomycetes	Planctomycetia	Gemmatales	Isosphaeraceae	Unidentified	Unidentified	0.987614	0.3812	0.4055	0.9783	35.7	34.7
Bacteria	Acidobacteria	Solibacteres	Solibacterales	Solibacteraceae	Candidatus Solibacter	Unidentified	0.999688	0.363	0.3861	1.364	13	40
Bacteria	Actinobacteria	Actinobacteria	Actinomycetales	Micrococcaceae	Arthrobacter	Unidentified	0.969133	0.3585	0.3813	1.746	5.67	45.7
Bacteria	Actinobacteria	Actinobacteria	Actinomycetales	Thermomonosporaceae	Actinoallomurus	Unidentified	0.85117	0.3194	0.3397	2.085	9.33	48.3
Bacteria	Actinobacteria	Actinobacteria	Actinomycetales	Nocardioidaceae	Nocardioides	Unidentified	0.992127	0.313	0.3329	2.418	10.3	41.7
Bacteria	Proteobacteria	Alphaproteobacteria	Rhizobiales	Hyphomicrobiaceae	Rhodoplanes	Unidentified	0.927295	0.2839	0.3019	2.72	0	34.7
Bacteria	Actinobacteria	Actinobacteria	Actinomycetales	Nocardioidaceae	Nocardioides	Unidentified	0.99302	0.2593	0.2758	2.996	0	31.7
Bacteria	Actinobacteria	Acidimicrobiia	Acidimicrobiales	Unidentified	Unidentified	Unidentified	0.966426	0.2238	0.238	3.234	0	27.3
Bacteria	Chloroflexi	TK10	B07_WMSP1	Unidentified	Unidentified	Unidentified	0.987757	0.2211	0.2351	3.469	0	27

b) The most affected bacterial communities between BP+R and control at Day 20 post inoculation in the BP experiment.

Kingdom	Phylum	Class	Order	Family	Genus	Species	Confidence	Av. dissim	Contrib. %	Cumulative %	Mean BP+R	Mean control
Bacteria	Firmicutes	Bacilli	Bacillales	Planococcaceae	Sporosarcina	Unidentified	0.758025	0.5168	0.554	0.554	10.3	64.3
Bacteria	Actinobacteria	Actinobacteria	Actinomycetales	Micrococcaceae	Arthrobacter	Unidentified	0.969133	0.3639	0.3901	0.9441	3.67	45.7
Bacteria	Actinobacteria	Actinobacteria	Actinomycetales	Nocardioidaceae	Nocardioides	Unidentified	0.992127	0.3412	0.3657	1.31	0	41.7

Bacteria	Acidobacteria	Solibacteres	Solibacterales	Solibacteraceae	Candidatus Solibacter	Unidentified	0.999688	0.3275	0.3511	1.661	0	40
Bacteria	Actinobacteria	Actinobacteria	Actinomycetales	Thermomonosporaceae	Actinoallomurus	Unidentified	0.85117	0.3139	0.3365	1.997	10	48.3
Bacteria	Planctomycetes	Planctomycetia	Gemmatales	Isosphaeraceae	Unidentified	Unidentified	0.987614	0.2839	0.3043	2.302	0	34.7
Bacteria	Proteobacteria	Alphaproteobacteria	Rhizobiales	Hyphomicrobiaceae	Rhodoplanes	Unidentified	0.927295	0.2839	0.3043	2.606	0	34.7
Bacteria	Actinobacteria	Actinobacteria	Actinomycetales	Nocardioidaceae	Nocardioides	Unidentified	0.99302	0.2802	0.3004	2.906	7.67	31.7
Bacteria	Actinobacteria	Actinobacteria	Actinomycetales	Micromonosporaceae	Micromonospora	Unidentified	0.883091	0.2448	0.2623	3.169	17.7	24
Bacteria	Actinobacteria	Actinobacteria	Actinomycetales	Streptomycetaceae	Streptomyces	Unidentified	0.90454	0.2347	0.2516	3.42	15	23.7

c) The most affected bacterial communities between BP+C and control at Day 43 post inoculation in the BP experiment.

Kingdom	Phylum	Class	Order	Family	Genus	Species	Confidence	Av. dissim	Contrib. %	Cumulative %	Mean BP + C	Mean control
Bacteria	Acidobacteria	Solibacteres	Solibacterales	Solibacteraceae	Candidatus Solibacter	Unidentified	0.999692	0.4954	0.5313	0.5313	60.5	0
Bacteria	Planctomycetes	Planctomycetia	Gemmatales	Isosphaeraceae	Unidentified	Unidentified	0.973174	0.4463	0.4786	1.01	54.5	0
Bacteria	Actinobacteria	Thermoleophilia	Solirubrobacterales		Unidentified	Unidentified	1	0.4135	0.4435	1.453	50.5	4.33
Bacteria	Actinobacteria	Actinobacteria	Actinomycetales	Thermomonosporaceae	Actinoallomurus	Unidentified	0.892564	0.389	0.4172	1.871	55.5	8
Bacteria	Proteobacteria	Alphaproteobacteria	Sphingomonadales	Sphingomonadaceae	Kaistobacter	Unidentified	0.998451	0.3808	0.4084	2.279	46.5	0
Bacteria	Chloroflexi	TK10	B07_WMSP1	Unidentified	Unidentified	Unidentified	0.985315	0.3767	0.404	2.683	46	14.3
Bacteria	Proteobacteria	Gammaproteobacteria	Xanthomonadales	Xanthomonadaceae	Lysobacter	Unidentified	0.92028	0.3685	0.3952	3.078	45	0
Bacteria	Actinobacteria	Thermoleophilia	Solirubrobacterales	Conexibacteraceae	Unidentified	Unidentified	0.891463	0.3603	0.3864	3.465	44	0
Bacteria	Proteobacteria	Betaproteobacteria	Burkholderiales	Oxalobacteraceae	Massilia	Unidentified	0.998298	0.3439	0.3689	3.834	42	0
Bacteria	Proteobacteria	Alphaproteobacteria	Rhizobiales	Hyphomicrobiaceae	Rhodoplanes	Unidentified	0.943398	0.3439	0.3689	4.202	42	0

Supplementary Table 2

a) The most affected protists communities between BP+R+C and control at Day 20 post inoculation in the BP experiment

Kingdom	Phylum	Class	Order	Family	Genus	Species	Confidence	Av. dissim	Contrib. %	Cumulative %	Mean BP+R+C	Mean control
Eukaryota	SAR	Rhizaria	Cercozoa	Guttulinupsidae	Rosculus	Unidentified	1	3.224	3.401	3.401	345	43.7
Eukaryota	SAR	Stramenopiles	Ochrophyta	Chromulinales	Poteriospumella	Unidentified	0.998831	1.977	2.085	5.486	184	0
Eukaryota	SAR	Stramenopiles	Ochrophyta	Chromulinales	Poteriospumella	Unidentified	0.998918	1.389	1.465	6.952	131	0
Eukaryota	SAR	Stramenopiles	Ochrophyta	Chromulinales	Poteriospumella	Unidentified	0.998867	1.334	1.407	8.359	0	125
Eukaryota	SAR	Unidentified	Unidentified	Unidentified	Unidentified	Unidentified	1	1.056	1.114	9.472	0	98.7
Eukaryota	SAR	Stramenopiles	Ochrophyta	Chromulinales	Poteriospumella	Unidentified	0.998846	0.9821	1.036	10.51	0	91.3
Eukaryota	SAR	Stramenopiles	Ochrophyta	Chromulinales	Poteriospumella	Unidentified	0.998482	0.9778	1.031	11.54	91.3	0
Eukaryota	SAR	Unidentified	Unidentified	Unidentified	Unidentified	Unidentified	1	0.9645	1.017	12.56	46.3	102
Eukaryota	SAR	Stramenopiles	Ochrophyta	Chromulinales	Poteriospumella	Unidentified	0.998755	0.9547	1.007	13.56	0	89.7
Eukaryota	SAR	Alveolata	Ciliophora	Hypotrichia	Unidentified	Unidentified	0.995708	0.9327	0.984	14.55	86.7	0

b) The most affected protists communities between BP+R and control at Day 20 post inoculation in the BP experiment

Kingdom	Phylum	Class	Order	Family	Genus	Species	Confidence	Av. dissim	Contrib. %	Cumulative %	Mean BP+R	Mean control
Eukaryota	SAR	Rhizaria	Cercozoa	Guttulinupsidae	Rosculus	Unidentified	1	1.95	2.071	2.071	227	43.7
Eukaryota	SAR	Stramenopiles	Ochrophyta	Chromulinales	Poteriospumella	Unidentified	0.998761	1.83	1.945	4.016	172	0
Eukaryota	SAR	Stramenopiles	Ochrophyta	Chromulinales	Poteriospumella	Unidentified	0.998867	1.33	1.413	5.429	0	125

Eukaryota	SAR	Stramenopiles	Ochrophyta	Chromulinales	Poteriospumella	Unidentified	0.998855	1.171	1.244	6.673	109	0
Eukaryota	SAR	Unidentified	Unidentified	Unidentified	Unidentified	Unidentified	1	1.052	1.118	7.791	0	98.7
Eukaryota	SAR	Stramenopiles	Ochrophyta	Chromulinales	Poteriospumella	Unidentified	0.99861	1.006	1.069	8.86	94.7	0
Eukaryota	SAR	Unidentified	Unidentified	Unidentified	Unidentified	Unidentified	1	0.9892	1.051	9.911	28.7	102
Eukaryota	SAR	Stramenopiles	Ochrophyta	Chromulinales	Poteriospumella	Unidentified	0.998846	0.9792	1.04	10.95	0	91.3
Eukaryota	SAR	Alveolata	Ciliophora	Unidentified	Unidentified	Unidentified	0.998846	0.9522	1.012	11.96	88.7	0
Eukaryota	SAR	Stramenopiles	Ochrophyta	Chromulinales	Poteriospumella	Unidentified	0.998755	0.9519	1.011	12.97	0	89.7

c) The most affected protists communities between BP and control at Day 3 post inoculation in the BP experiment

Kingdom	Phylum	Class	Order	Family	Genus	Species	Confidence	Av. dissim	Contrib. %	Cumulative %	Mean BP	Mean control
Eukaryota	SAR	Stramenopiles	Ochrophyta	Chromulinales	Poteriospumella	Unidentified	0.998846	1.964	2.506	2.506	86.7	139
Eukaryota	SAR	Stramenopiles	Ochrophyta	Chromulinales	Poteriospumella	Unidentified	0.998867	1.698	2.167	4.673	99.7	114
Eukaryota	SAR	Stramenopiles	Ochrophyta	Chromulinales	Poteriospumella	Unidentified	0.998755	1.527	1.949	6.621	96.3	99.7
Eukaryota	SAR	Alveolata	Ciliophora	Hypotrichia	Unidentified	Unidentified	0.995728	1.022	1.304	7.925	46.3	73.3
Eukaryota	SAR	Alveolata	Ciliophora	Unidentified	Unidentified	Unidentified	0.998819	0.9647	1.231	9.156	75.3	23.7
Eukaryota	SAR	Alveolata	Ciliophora	Hypotrichia	Unidentified	Unidentified	0.995727	0.9067	1.157	10.31	46.3	62.3
Eukaryota	SAR	Alveolata	Ciliophora	Hypotrichia	Gonostomum	Unidentified	0.703033	0.8636	1.102	11.42	42	61
Eukaryota	SAR	Alveolata	Ciliophora	Unidentified	Unidentified	Unidentified	0.998908	0.8275	1.056	12.47	53	54
Eukaryota	SAR	Alveolata	Ciliophora	Hypotrichia	Unidentified	Unidentified	0.802934	0.7881	1.006	13.48	54.3	41
Eukaryota	SAR	Alveolata	Ciliophora	Hypotrichia	Unidentified	Unidentified	0.995458	0.7636	0.9744	14.45	53.3	37.7

d) The most affected protists communities between BP+R+C and control at Day 43 post inoculation in the BP experiment

Kingdom	Phylum	Class	Order	Family	Genus	Species	Confidence	Av. dissim	Contrib. %	Cumulative %	Mean BP+R+C	Mean control
Eukaryota	SAR	Stramenopiles	Ochrophyta	Chromulinales	Poteriospumella	Unidentified	0.998918	1.984	2.11	2.11	180	0
Eukaryota	SAR	Rhizaria	Cercozoa	Guttulinupsidae	Rosculus	Unidentified	1	1.254	1.334	3.444	34	128
Eukaryota	SAR	Stramenopiles	Ochrophyta	Chromulinales	Poteriospumella	Unidentified	0.998846	1.242	1.321	4.765	0	113
Eukaryota	SAR	Rhizaria	Cercozoa	Cercomonadidae	Cercomonas	Unidentified	1	1.228	1.306	6.071	174	78.3
Eukaryota	SAR	Stramenopiles	Ochrophyta	Chromulinales	Poteriospumella	Unidentified	0.998867	1.155	1.229	7.3	0	103
Eukaryota	SAR	Stramenopiles	Ochrophyta	Chromulinales	Poteriospumella	Unidentified	0.998755	1.094	1.163	8.463	0	100
Eukaryota	SAR	Stramenopiles	Ochrophyta	Chromulinales	Poteriospumella	Unidentified	0.998831	1.048	1.115	9.578	94	0
Eukaryota	SAR	Unidentified	Unidentified	Unidentified	Unidentified	Unidentified	1	0.9964	1.06	10.64	90.7	0
Eukaryota	SAR	Alveolata	Ciliophora	Hypotrichia	Unidentified	Unidentified	0.995727	0.9793	1.042	11.68	0	89.3
Eukaryota	SAR	Stramenopiles	Ochrophyta	Chromulinales	Poteriospumella	Unidentified	0.998482	0.9786	1.041	12.72	90	0

e) The most affected protists communities between BP+R and control at Day 43 post inoculation in the BP experiment

Kingdom	Phylum	Class	Order	Family	Genus	Species	Confidence	Av. dissim	Contrib. %	Cumulative %	Mean BP+R	Mean control
Eukaryota	SAR	Rhizaria	Cercozoa	Guttulinupsidae	Rosculus	Unidentified	1	1.729	1.85	1.85	227	78.3
Eukaryota	SAR	Unidentified	Unidentified	Unidentified	Unidentified	Unidentified	1	1.444	1.546	3.396	88.7	128
Eukaryota	SAR	Stramenopiles	Ochrophyta	Chromulinales	Poteriospumella	Unidentified	0.998846	1.242	1.329	4.725	0	113
Eukaryota	SAR	Stramenopiles	Ochrophyta	Chromulinales	Poteriospumella	Unidentified	0.998867	1.155	1.236	5.961	0	103
Eukaryota	SAR	Stramenopiles	Ochrophyta	Chromulinales	Poteriospumella	Unidentified	0.99861	1.117	1.195	7.156	100	0

Eukaryota	SAR	Stramenopiles	Ochrophyta	Chromulinales	Poteriospumella	Unidentified	0.998855	1.103	1.18	8.336	102	0
Eukaryota	SAR	Stramenopiles	Ochrophyta	Chromulinales	Poteriospumella	Unidentified	0.998755	1.094	1.17	9.507	0	100
Eukaryota	SAR	Stramenopiles	Ochrophyta	Chromulinales	Poteriospumella	Unidentified	0.998761	1.032	1.104	10.61	93.3	0
Eukaryota	SAR	Alveolata	Ciliophora	Hypotrichia	Unidentified	Unidentified	0.995727	0.9791	1.048	11.66	0	89.3
Eukaryota	SAR	Unidentified	Unidentified	Unidentified	Unidentified	Unidentified	1	0.8354	0.8939	12.55	77	0

a) The most affected protists communities between BP+C and control at Day 43 post inoculation in the BP experiment

Kingdom	Phylum	Class	Order	Family	Genus	Species	Confidence	Av. dissim	Contrib. %	Cumulative %	Mean BP+C	Mean control
Eukaryota	SAR	Rhizaria	Cercozoa	Cercomonadidae	Cercomonas	Unidentified	1	1.819	1.959	1.959	254	102
Eukaryota	SAR	Stramenopiles	Ochrophyta	Chromulinales	Poteriospumella	Unidentified	0.998867	1.34	1.444	3.403	0	125
Eukaryota	SAR	Alveolata	Ciliophora	Hypotrichia	Unidentified	Unidentified	0.995721	1.121	1.208	4.611	106	0
Eukaryota	SAR	Unidentified	Unidentified	Unidentified	Unidentified	Unidentified	1	1.061	1.143	5.753	0	98.7
Eukaryota	SAR	Stramenopiles	Ochrophyta	Chromulinales	Poteriospumella	Unidentified	0.998866	1.019	1.097	6.851	96.3	0
Eukaryota	SAR	Stramenopiles	Ochrophyta	Chromulinales	Poteriospumella	Unidentified	0.998846	0.987	1.063	7.914	0	91.3
Eukaryota	SAR	Stramenopiles	Ochrophyta	Chromulinales	Poteriospumella	Unidentified	0.998732	0.9866	1.063	8.977	92.7	0
Eukaryota	SAR	Stramenopiles	Ochrophyta	Chromulinales	Poteriospumella	Unidentified	0.998755	0.9594	1.033	10.01	0	89.7
Eukaryota	SAR	Stramenopiles	Ochrophyta	Chromulinales	Poteriospumella	Unidentified	0.998862	0.9196	0.9906	11	83.3	0
Eukaryota	SAR	Unidentified	Unidentified	Unidentified	Unidentified	Unidentified	1	0.9094	0.9796	11.98	0	85
